# Differences in Moral Judgment on Animal and Human Ethics Issues between University Students in Animal-Related, Human Medical and Arts Programs

**DOI:** 10.1371/journal.pone.0149308

**Published:** 2016-03-02

**Authors:** Joy M. Verrinder, Remo Ostini, Clive J. C. Phillips

**Affiliations:** 1 Centre for Animal Welfare and Ethics, School of Veterinary Science, University of Queensland, Gatton, Queensland 4343, Australia; 2 Rural Clinical School Research Centre, School of Medicine, University of Queensland, Toowoomba, Australia; Harvard University Faculty of Arts and Sciences, UNITED STATES

## Abstract

Moral judgment in relation to animal ethics issues has rarely been investigated. Among the research that has been conducted, studies of veterinary students have shown greater use of reasoning based on universal principles for animal than human ethics issues. This study aimed to identify if this was unique to students of veterinary and other animal-related professions. The moral reasoning of first year students of veterinary medicine, veterinary technology, and production animal science was compared with that of students in non-animal related disciplines of human medicine and arts. All students (n = 531) completed a moral reasoning test, the VetDIT, with animal and human scenarios. When compared with reasoning on human ethics issues, the combined group of students evaluating animal ethics issues showed higher levels of Universal Principles reasoning, lower levels of Personal Interest reasoning and similar levels of Maintaining Norms reasoning. Arts students showed more personal interest reasoning than students in most animal-related programs on both animal and human ethics issues, and less norms-based reasoning on animal ethics issues. Medical students showed more norms-based reasoning on animal ethics issues than all of the animal-related groups. There were no differences in principled reasoning on animal ethics issues between program groups. This has implications for animal-related professions and education programs showing that students’ preference for principled reasoning on animal ethics issues is not unique to animal-related disciplines, and highlighting the need to develop student (and professional) capacity to apply principled reasoning to address ethics issues in animal industries to reduce the risk of moral distress.

## Introduction

Moral judgment has been identified as a cognitive development process through three levels of reasoning—preconventional (based on avoidance of punishment and satisfaction of personal interests), conventional (based on a desire to maintain society’s laws and institutional rules) and post-conventional (based on universal ethical principles of justice and impartiality for the welfare of all individuals) [1]. To investigate moral judgment development, Kohlberg used a Moral Judgment Interview in which respondents discussed their concerns in relation to specific human ethics issues. A 20 year longitudinal study found moral judgment development to be positively correlated with age, socio-economic status, IQ, and education [2]. Other tests have been developed to simplify the process of assessing levels of moral judgment e.g. Gibb’s Sociomoral Reflection Measure [3] and Rest’s Defining Issues Test (DIT), the latter being used extensively in higher education and professional contexts [4]. While Kohlberg’s highest stage of moral development was focussed on justice, Rest’s is a broader definition which encompasses all ethical theories for organising cooperation in society that are based on:

- The primacy of moral criteria in which conventions are alterable with duties and rights following from the moral purpose
- Appeal to a positive and constructive ideal incorporating the greatest good for all, guaranteeing minimal rights and protection for everyone, engendering caring, and mandating fairness
- Sharable ideals that are not self-serving at the expense of others, that respect others, and are not shielded by a privileged source of authority not subject to scrutiny
- Full reciprocity which requires that social norms are not biased in favour of some at the expense of others and rely on consensus based on ideals and logical coherence rather than established practice and existing authority

Moral theories that advocate that morality is nothing but the personal expression of approval or disapproval, that cooperation is a bad idea, or that are based on strict adherence to fundamentalist religious views not subject to scrutiny are excluded [4].

Studies conducted to identify moral judgment development of students in different professions have used human ethics issues such as whether one should steal to feed one’s family during a famine. A review of 33 moral judgment studies (6600 respondents) in medicine, dentistry, law and veterinary medicine confirmed that professional education programs do not promote moral judgment development unless the program contains a well-validated ethics curriculum [5]. However, three studies comparing first and final year veterinary students to identify impact of age/education on moral judgment development showed mixed results. One of these, a large study, using the DIT as the moral judgment measure, of first and fourth year veterinary student volunteers (n = 98) demonstrated similar mean universal principles (UP) scores at the beginning and end of the four year veterinary medicine course [6]. An earlier pilot study (n = 20) using the Moral Judgment Interview found similar results [7]. The third study of 57 students showed an increase during the course, using Gibbs’ Socio-moral Reflection Measure.[8]First year medical students have shown higher levels of moral judgment scores on DIT human ethics scenarios (mean UP score of 51) than college students generally (mean UP score of 46), but lower than philosophy students (mean UP score of 64) [9]. In DIT studies, formal education has had the most significant effect on UP scores, more than age, socio-economic status, region of country, sex, religion or profession, and UP scores tend to plateau at the highest level of a person’s formal education [4].

Very few studies have been done to assess moral judgment in relation to animal ethics issues. With an expansion of intensive use of animals worldwide[10], increasing knowledge of animals’ capacities and sentience[11–13], changes in relationships with companion animals, and the growing interest of society[14], the veterinary profession has become increasingly aware of the need to be skilled in ethical decision-making in relation to animals’ welfare and treatment [15, 16]. The World Health Organisation and governments have engaged veterinarians to develop policy and assist animal industries to develop better health, welfare and ethical practices in the various uses of animals. In many jurisdictions, animal research and teaching using animals can only be conducted with the approval of an animal ethics committee, which often includes a veterinarian. Yet there are currently no consistent international competencies required for moral judgment development in veterinary and other animal science courses and little is known about how veterinarians reason in relation to animal ethics issues.

To address this gap in knowledge, a test to identify moral judgment development in relation to animal ethics issues experienced by veterinarians, the VetDIT, based on Rest’s Defining Issues Test (DIT)[4], was developed and piloted in 2012 [17]. The VetDIT includes three animal ethics issues and, for comparison, three human ethics issues from the DIT. This study showed that while veterinary students in the first year of their university program had similar reasoning levels to US Freshmen on human ethics issues, they had lower Personal Interest (PI) and higher Universal Principles (UP) reasoning scores on animal than on human ethics issues. It was considered that this could be due to the three animal issues presenting greater potential suffering than the three human scenarios, or because of students’ desire to help animals, demonstrated through their choice of an animal-related career. In an Australian study, 70% of students indicated “helping sick or injured animals” and 40% indicated “improving the way animals are treated” in their top three motivators for studying veterinary medicine [18]. In the first VetDIT version, there was an extra UP option in two of the three scenarios which may have increased the chance of UP items being ranked as important. This was addressed with the refinement and validation of a revised VetDIT- Version 2 (V2) in which the scenarios and questions were simplified and clarified, and the number of PI, MN and UP items balanced across the three scenarios.

The aim of this study was to use the revised VetDIT-V2 to compare moral judgment development in relation to animal ethics issues of students of animal related disciplines i.e. veterinary medicine, veterinary technology, animal science with non-animal related disciplines i.e. human medicine and arts students. Because previous research has shown the majority of veterinarians choose their course to help animals [18], it was hypothesised that veterinary students may use more principled reasoning on animal ethics issues, than students of animal science whose focus was largely animal farming, human-focussed medicine and a general ethics course grounded in moral philosophy with only one lecture on the ethics of animal experimentation. It was also hypothesized that arts students studying an ethics course may show more principled reasoning, particularly on human ethics issues, as they would be more aware of ethics theory.

## Materials and Methods

### Participants

A total of 531 first year students from five courses at the University of Queensland, Australia completed the VetDIT Version 2 and were retained after standardised reliability checks (based on inconsistencies between items rated and ranked, missing data, selection of meaningless items, and indiscriminate answers) [19]. Three groups were from animal-related programs, and two from non-animal programs, as follows:

Animal-related programs

- 130 first-year Bachelor of Veterinary Science (Vet Sci) students (88% of the cohort) in their second semester, prior to animal ethics teaching, although 35 students (27% of the respondents) had a previous degree in which they may have had some ethics teaching;
- 65 1^st^ Yr Bachelor of Applied Science—Veterinary Technology (Vet Tech) students (55% of the cohort) in their second semester with no previous ethics teaching in their course, although 5 students (6%) had previous degrees which may have included some ethics teaching;
- 191 first year Bachelor of Applied Science—Animal Science (Anim Sci) students (55% of the cohort) in their first semester, with 52% completing the test prior to two hours of ethics lectures, and 48% post teaching. No adjustment was made to the post test scores as these lectures had no significant effect on their DIT scores [20]. Some 13 students (7% of respondents) had a previous degree which may have included some ethics teaching.

Non-animal related programs

- 95 first year Bachelor of Medicine, Bachelor of Surgery students (Med) (21% of the cohort) at the beginning of their course with no medical ethics teaching. However all these students had completed a previous degree and may therefore have had some ethics teaching.
- 50 first year Bachelor of Arts (Arts) students (49% of the cohort) in the last three weeks of an Introduction to Ethics course.

### Procedures

Written approval for this study was obtained from the University of Queensland Behavioural and Social Sciences Ethical Review Committee. It included students’ providing written consent for their DIT response to be used for research purposes, by recording a unique ID based on a provided formula on their DIT response, also enabling anonymity and confidentiality. Students completed the VetDIT in one 50 minute session. The test was incorporated into the teaching programs for Vet Sci, Anim Sci, Vet Tech and Arts students, and for the first two was accessible electronically on the University’s teaching portal [21] for those attending or unable to attend the session. Med students were invited to participate following a one hour session on research opportunities and offered an incentive to participate by being eligible for a draw in a cash prize of $100.

### Materials

The VetDIT Version 2 [20] is based on the Defining Issues Test (DIT-2) [22], which uses Kohlberg's six hierarchical stages in three developmental levels of moral judgment but redefines them as three schemas i.e. general cognitive structures which are applied to help understand new information:

- Schema 1 Personal Interest (PI)—recognition of authority and reciprocal relationships which result in reward or punishment for the person
- Schema 2 Maintaining Norms (MN)—abiding by existing rules and regulations set by governments or professional groups.
- Schema 3 Post-conventional, referred to here as Universal Principles (UP), emphasising moral ideals which are constructive and not self-serving at the expense of others.

Development occurs through adoption of higher level schemas. However, unlike Kohlberg’s interpretation where progress occurs through one stage at a time, in the DIT, people may utilise more than one schema in their reasoning, and there may be cross-cultural variations [4].

The VetDIT V2 includes three animal ethics scenarios: Euthanasia of a healthy dog, Reporting of sub-standard pig husbandry, and Breeding modification of hens. Three of the five human scenarios in Rest et al’s DIT-2 [22] were included for comparison: Stealing during a famine, Reporting previous criminal history of a government candidate, and Cancelling a school meeting due to violence in previous meetings. Each animal scenario has 12 questions with three or four questions representing each of the different levels of reasoning i.e. Personal Interest, Maintaining Norms or Universal Principles (UP based on a mixture of deontological, utilitarian, care or virtue ethics frameworks), plus one meaningless item for validity testing. Across the three scenarios, eleven questions represent each of PI, MN and UP reasoning. The VetDIT V2 is provided in S1 Appendix. Anyone wanting to use the test should contact us to see if there are revised versions and for the scoring process. Students initially rate each question according to how important they consider it to be when making a decision about what to do in each scenario. Students then rank the four questions they consider most important. These rankings are then scored, with 4 for the highest ranked, reducing to 1 for the fourth ranked question. These scores are allocated to PI, MN or UP based on which schema each ranked question represents. Each schema’s total scores for the three animal and three human scenarios are converted to percentages.

To identify the importance given to different ethical frameworks within UP i.e. deontological, utilitarian, care and virtue ethics, ranked scores were tallied for each of these frameworks using the same scoring system, e.g. if a deontological question “Does the dog have a right to life?” was ranked as most important, 4 points; ranked second, 3 points; ranked third, 2 points; and ranked fourth, 1 point. The summated points for each ethical framework were compared to identify students’ priorities when making decisions on animal ethics issues.

Validation of the VetDIT is ongoing. However studies so far have shown that it is sensitive to interventions designed to improve moral reasoning, and differentiates groups which one would expect to have greater expertise i.e. students with a previous degree (20).

### Demographics

Demographic information was gathered when completing the VetDIT, that is, students’ age, sex, previous university degrees, which degrees, whether English was their primary language and experience with companion animals, farm animals and horses. Out of the 531 students, one student did not provide information on their previous degree, and two students on whether English was their primary language.

### Statistical Analysis

Minitab Statistical Software (Version 16. State College, PA: Minitab Inc) was used to analyse the data. A general linear model was used to identify effects on PI, MN and UP DIT scores, of program, age, sex, previous degree, language, and experience with companion animals, farm animals (e.g. pigs, hens) and horses. Residuals were tested for normal distribution using the Anderson-Darling test. Universal Principles (UP) residuals for human scenarios were normally distributed and a General Linear model was used (with least square means) to identify program and demographic effects. MN Human and UP residuals for animal scenarios approximated a normal distribution, and PI residuals for both human and animal scenarios (P = 0.006 and <0.005) and MN animal residuals were not normally distributed, even after a variety of transformations. The Mood’s Median Test was therefore used to identify program and demographic effects on PI, MN and UP animal scenarios and PI and MN human scenarios. Program effects for these scenarios and differences between human and animal scores for PI, MN and UP were further identified using Mann-Whitney and Tukey’s pairwise comparisons. A regression analysis was used to identify the effect of age on UP human reasoning.

Correlations of PI, MN and UP scores between individual scenarios and between the combined three human and combined three animal scenarios were obtained using Spearman ranked data because the residual distributions were not normal by the Anderson-Darling test. Differences in the variation in PI, MN and UP scores between courses and for animal and human scenarios was analysed using coefficients of variation (CV) across individuals within courses, with CV for human and animal PI, MN and UP compared by a general linear model with 5 replicates being the CV for each course. Residuals were normally distributed by the Anderson Darling test. Comparison between human and animal scenarios was not possible by a general linear model as residuals were not normally distributed, so a Moods median test was used. Variation in scores between the six individual scenarios was also analysed by coefficients of variation across individual scores, using a general linear model as residuals were normally distributed by the Anderson Darling test.

## Results

### Demographic characteristics

Of the five groups of student respondents, Med students had the highest median age, and Arts students had the largest age range (Table 1). Students within the animal-related courses were predominantly female, while almost half in the ethics group and more than half in the Med group were male. All Med students had previous degrees, in contrast with just 27% of Vet Sci students, and less than ten percent of all other animal related courses. English was the primary language for the majority of students in all groups. Med students had the least exposure to companion animals, farm animals and horses. In the animal-related courses, Vet Tech and Anim Sci students reported that they had greater experience than Vet Sci students with companion animals, farm animals and horses.

**Table 1:** Number(%) of 1^st^ Year Vet Sci, Vet Tech, Bachelor of Applied Science (Anim Sci) students, and 3^rd^ Year Veterinary Students by age range, median age, age group, sex, previous degree, English as primary language, and experience with companion animals, farm animals and horses.

Demographics	ArtsN = 50	Vet Sci N = 130	Vet Tech N = 65	Anim Sci N = 191	Med N = 95
**Age**					
Range	16–61	17–42	17–32	16–50	20–36
Standard Error of Mean	0.893	0.329	0.339	0.316	0.342
Median	18	20	18	18	23
No (%)< 21	46 (91)	76 (58)	57 (88)	145 (76)	14 (15)
No (%)21-25	1(2)	45 (35)	6 (9)	29 (15)	59 (62)
No (%)>25	3(6)	9 (7)	2 (3)	17 (10)	22 (23)
**No (%)Female**	28 (56)	108 (83)	62 (95)	168 (88)	39 (41)
**No (%)Previous Degree**	2 (4)	35 (27)	5 (8)	13 (7)	95 (100)
**No (%)English as primary language**	48 (96)	112 (86)	63 (98)	179 (94)	89 (94)
**No (%)Very great or great experience / minimal or no experiencewith:**					
Companion Animals	38 (76)/7 (14)	92(71)/13(10)	55 (85)/6 (9)	163 (85)/13 (7)	58 (61)/18 (19)
Farm Animals	13 (26)/22 (44)	23(18)/74(57)	18 (28)/25 (38)	62 (32)/66 (35)	13 (14)/57 (60)
Horses	9 (18)/26 (52)	32(25)/74(57)	24 (37)/30 (46)	83 (43)/70 (37)	15 (16)/68 (72)

### Comparison of Animal and Human Scores

Comparing scores on animal (n = 531; median PI 3.4, median MN 34.5, mean UP 62.7) and human scenarios (median PI 28.1, median MN 31.6, mean UP 38.0), the animal scenarios had lower PI (p<0.001), similar MN (p = 0.27) and higher UP scores (p<0.001).

### Program Effects

On animal issues, Arts students had higher levels of PI reasoning than Anim Sci and Med students (p. < 0.05; see Table 2). Vet Sci and Vet Tech students had higher levels of PI reasoning than Med students, and were similar to Anim Sci students. Arts students had lower MN reasoning scores than Med, Vet Sci and Vet Tech students, but not Anim Sci students. Vet Sci, Vet Tech and Anim Sci students’ MN reasoning scores on animal issues were lower than Med students' scores. There was no effect of program on UP reasoning for animal issues. On human issues, Arts students had higher PI reasoning scores than Vet Sci, Anim Sci and Med students, but not Vet Tech students. Med and Vet Sci students had higher UP scores than Anim Sci students. There was no effect of program of study on MN reasoning for human issues (Table 2).

**Table 2:** Personal Interest (PI), Maintaining Norms (MN) and Universal Principles (UP) scores for animal and human scenarios for students of Bachelor of Arts (Arts), medicine/surgery (Med), applied science (Anim Sci), veterinary science (Vet Sci) and veterinary technology (Vet Tech).

ReasoningType	Course					Previous Degree	No PreviousDegree	Male	Female	English Primary Language	English Not Primary Language	P ValueCourse	P ValuePrevious Degree	P ValueSex	P Value Language
	Arts	Med	AnimSci	Vet Sci	VetTech										
**Animal** **PI, median**	6.9^a^	0.0^c^	3.4 ^b^^,^^c^	3.4^b^	6.9^a^^b^	0.00	3.4	3.4	3.4	3.4	3.4	0.004	0.03	0.12	0.42
**MN, median**	24.1^c^	37.9^a^	31.0 ^b^^c^	32.8^b^	34.5^b^	36.2	31.0	37.9	31.0	31.0	37.9	0.001	0.08	0.03	0.02
**UP, median**	65.5	58.6	65.5	65.5	62.1	62.1	65.5	60.3	65.5	65.5	55.2	0.11	0.56	0.001	0.06
**Human****PI, median**	35.1^a^	24.6^b^	28.1^b^	27.2^b^	28.1^a^	24.6	28.1	29.8	28.1	28.1	28.1	0.05	0.58	0.05	0.76
**MN, median**	28.1	31.6	35.1	29.8	35.1	31.6	31.6	31.6	31.6	31.6	33.3	0.30	0.18	0.85	0.85
**UP, mean**	36.0^a^,^b^	43.6^a^	34.4^b^	41.5^a^	35.8^a^^,^^b^	37.4	39.1	35.3	41.2	39.1	37.4	0.004	0.57	0.002	0.56

^a,b,c^ Medians and means with common superscripts do not differ significantly (p>0.05). For parameters tested by Moods Median Test, pairwise comparisons are by Mann-Whitney Test; for parameters tested by General Linear Model, pairwise comparisons are by Tukey’s Multiple Comparison Test

### Other Demographic Effects

For animal ethics issues, males had higher MN and lower UP reasoning than females (Table 2). Students with a previous degree had lower PI scores and there was a trend for higher MN scores than those with no previous degree. Students whose English was not their primary language had higher MN reasoning, and there was a trend for lower UP reasoning, than for those whose primary language was English.

For human ethics issues, males had higher PI and lower UP reasoning scores than females. Age had a large effect on UP reasoning scores on human scenarios, with UP scores increasing rapidly with age, although the r^2^ value was low: UP (Human) = 28.8 (+ 3.06) +0.45 (+ 0.144) Age, R-Sq = 1.8%, p = 0.002. There was no significant effect of experience with companion animals, farm animals or horses on PI, MN or UP reasoning for either animal or human scenarios (P > 0.10).

### Importance of different ethical frameworks in UP judgment on animal ethics issues

The weighted scores for different ethical frameworks used as the basis for UP questions, in order of importance in each scenario, were: *Euthanasia scenario*: deontological (right to life) 1563, utilitarian 875, care ethics 576, deontological (defy law to respect life) 387; *Pig husbandry scenario*: deontological 1490, utilitarian 893 and care ethics 571; *Breeding blind hens scenario*: Utilitarian 1450, Deontological (fairness) 1159, deontological (bodily integrity) 512, virtue ethics 148. Thus in the Euthanasia and Pig Husbandry scenarios, students prioritised deontological considerations of the animals’ right to life (euthanasia scenario) and treatment (pig husbandry scenario), over utilitarian, care and virtue ethics frameworks. In the breeding modification scenario, the deontological principle of fairness was second in importance to utilitarian considerations of weighing benefits and harms. Other deontological perspectives were of relatively low importance, i.e. in the euthanasia scenario, secretly rehoming the dog out of respect for its life; in the breeding modification scenario, respect for the bodily integrity of the hens.

### Relationships between combined human and animal scenarios and between individual scenarios

Animal and human scores were correlated within the PI, MN and UP schemas: the correlations coefficients (CC) of the ranked combined animal PI, MN and UP with combined human PI, MN and UP scores, respectively, were as follows: PI CC 0.20, P < 0.001; MN CC 0.17, P < 0.001; UP CC 0.15, P = 0.001.

PI, MN and UP scores for animal scenarios were all correlated between scenarios. Within schemas, the moral reasoning scores for the animal euthanasia scenario were highly correlated with those for the pig husbandry and breeding modification scenarios for MN (correlation coefficients CC 0.51, p<0.001 and CC 0.44, p<0.001) and UP (CC 0.56, p<0.001) and CC 0.55, p<0.001). For PI they were less highly correlated: PI (CC 0.19, p<0.001 and CC 0.14, p = 0.001 respectively). The scores for the pig husbandry scenario were also correlated with the breeding modification scenario again more for MN (CC 0.54, p<0.001), and UP (CC 0.59, p<0.001) than for PI (CC 0.27, p<0.001).

There were some low but significant correlations between animal and human scenarios. These included the animal euthanasia scores being correlated with famine scores for MN (CC 0.12, p = 0.005) and UP (CC 0.10, p = 0.02) scores, but not PI scores (CC 0.05, p = 0.22), and with the school meeting scenario for PI (CC 0.13, p = 0.002) and UP (CC 0.11, p = 0.013) but not MN scores (CC -0.002, p = 0.96). The reporter scenario was not correlated with the animal euthanasia scenario. The pig husbandry scenario scores were not correlated with the famine or reporter scenario scores, but were correlated with the school meeting scenario scores for PI (CC 0.12, p = 0.007) and UP (CC 0.13, p = 0.003) but not MN (CC 0.02, p = 0.60). The breeding modification scenario scores were not correlated with the famine, reporter or school board scenarios scores except for the school board UP scores (CC 0.13, p = 0.002).

Low correlations between human scenarios included the famine scenario being correlated with the reporter scenario for PI scores (CC 0.20, p<0.001), the school meeting scenario for MN scores (CC 0.12, p = 0.005) and the reporter and school meeting scenarios for UP scores (CC 0.23, p<0.001 and CC 0.10, p = 0.02 respectively). The reporter scenario scores also correlated with the school meeting scenarios for PI (CC 0.10, p = 0.02) and UP scores (CC 0.18, p<0.001) but not MN scores (0.06, p = 0.16).

### Variation in combined human and animal scenarios and in individual scenarios

There were no significant differences between courses in the coefficient of variation (henceforth variation) in PI, MN and UP scores (P > 0.10). Animal scenarios had much greater variation within a course than human scenarios (mean inter-quartile range [Q3-Q1] of CV for PI, MN and UP: animal = 79.5, human = 7.8). For animal scenarios variation in PI> MN> UP, whereas all three were similar for human scenarios (CV animal PI 126.2, MN 44.9, UP 26.2; human PI 47.5, MN 45.8, UP 41.8, SED 2.82, P < 0.001).

## Discussion

This research supports a previous study [17] finding that veterinary students prioritised principled reasoning when making decisions about animal ethics issues, more so than when reasoning about human ethics issues. However, it provides new evidence that predominantly principled reasoning on animal ethics issues is not unique to students of Vet Sci, despite their program choice being based on a desire to work with and help sick and injured animals [18]. Regardless of professional interest, when considering animal scenarios, principled reasoning was prioritised by both animal-related (Vet Sci, Vet Tech, Anim Sci) and non-animal related fields (Med and Arts), more so than personal gain and obedience to authority (PI), or compliance with existing laws and policies (MN).

Across the whole sample, the median PI and MN moral reasoning scores on the combined human scenarios, including only those students without a previous degree (n = 382; PI 28.07, MN 32.39,), were similar to mean scores of a mixed sample of US college freshmen across a range of disciplines and universities, gathered from 176 data sets (n = 2096; PI 28.5, MN 33.6) [23] and the mean UP score (38.6) was higher than for US Freshmen (32.3). The difference in UP scores of the US Freshmen group could be due to variability in moral judgment that has been found to exist between different types of universities and regions within the US. First-year Med students, all of whom had a previous degree, had higher median PI scores and similar median MN and mean UP scores on combined human scenarios (n = 95; PI 24.6, MN 31.6, UP 43.6), compared with mean scores of US professional degree students (n = 1582; PI 19.8, MN 31.4, UP 44.9) [23]. Differences in PI scores may have been due to different professional degrees. Also, as it was not clear at what stage the US students were in their professional degrees, and may have completed their professional degrees, the positive effects of education/age on moral reasoning [4], may have resulted in lower PI scores than for first year Australian medical students, although higher UP scores would then also be expected.

The study also suggests that first year students most often prioritise deontological reasoning over utilitarian, care and virtue ethics frameworks. The highest level of importance was given to the principle of the right to life in the euthanasia of a healthy dog scenario and the right of pigs to treatment in the poor husbandry scenario. The principle of fairness was a close second in importance in the breeding modification scenario (i.e. “Is it fair to manipulate animals to fit production systems?”), with greatest priority given to utilitarian reasoning i.e. weighing up the harms of existing intensive farming practices such as debeaking of hens, against breeding blind hens. Previous studies have shown that first year veterinary students at the University of Queensland support veterinary medicine requiring a commitment to animals’ interests over the interests of their owners/caregivers [18]. Further studies are needed to determine if first year students in various disciplines at other universities also prioritise deontological reasoning. However students’ motivation to take personal risks to protect an animals’ life seems questionable, based on the low priority given to: “Should the veterinarian secretly rehome the dog out of respect for its life?” in the euthanasia scenario. As well, very few students prioritised the right to bodily integrity i.e. “Is it disrespectful to interfere with the ‘wholeness’ of a bird?” with more importance being given to consideration of the extent of suffering than the comfort and pleasure from the birds’ sense of sight.

This prioritising of principled judgment on animal ethics issues, particularly the right to life and treatment, fairness, and weighing up the benefits and harms to all involved has implications for professional practice. Many animal-related professionals routinely engage in practices that restrict the welfare of animals within their care. Some have argued that medical [24], legal [25] and veterinary [26] professionals face challenges in living up to moral ideals because systems around them are dominated by personal interests, commercialism, and conventional morality. A moral climate of disillusionment and cynicism about the possibility of applying the ideals of postconventional moral reasoning in real life situations may result in inhibiting moral judgment [27] and moral motivation to apply these ideals. Despite having a professional degree, practicing veterinarians have been shown to have similar moral judgment scores to the general public on human ethics issues, and show no improvement with years of experience [28]. Further studies are needed to assess practising veterinarians’ moral judgment in relation to animal ethics issues.

Historically, the growth of the veterinary profession seems to have been based on PI reasoning, with a need to keep animals healthy to maximise usefulness i.e. fit and healthy horses used for power, transport and war, and animals farmed for food free from disease to raise productivity and support human population growth [29]. Following a major foot-and-mouth disease outbreak, and the need for more consistency in veterinary standards to keep animals healthy and useful, formalisation and regulation of the veterinary profession occurred in the UK from 1844 [29]. Veterinary associations have tended to use legislated norms (MN reasoning) as the basis for policies and positions on animal ethics issues. The Australian Veterinary Association’s Code of Professional Conduct requires its members to “always consider the health, welfare and respectful treatment of animals” and “understand and comply with all relevant laws and guidelines, especially those regarding animal welfare, veterinary client confidentiality, and the prescribing of restricted substances”[30]. These two requirements reflect conflicting demands between principled and maintaining norms reasoning. The code currently does not encourage leadership in developing or promoting laws and standards which apply universal ethical principles to decisions on animal ethics issues. This mismatch with current students’ prioritisation of principled reasoning is likely to contribute to moral distress "when one knows the right thing to do, but institutional or other constraints make it difficult to pursue the desired course of action” (Jameton cited in Raines) [31]. Moral distress has also been identified when moral decisions are followed, but in doing so they clash with legal regulations [32]. One way of addressing moral distress has been for the organisational culture to facilitate moral shift, in which the responsibility of, for example, killing healthy animals in a shelter, vet clinic or for medical training is shifted from the medical personnel to the animal owners who are seen as neglectful and irresponsible (Arluke cited in Scotney) [33] or to the those in authority in the organisation, such as the owners of the clinic [34] or the pound that provided the animals [35]. Other coping behaviours include overcompensating with or distancing from patients, and leaving the profession [31]. None of these resolve the ethical issues.

A universal principles approach to animal ethics education may therefore provide a unifying international objective for veterinary ethics education. Some teachers of veterinary ethics have taken a pluralist approach, encouraging students to identify their own personal perspective and promoting tolerance of a range of societal perspectives on how animals should be treated [36]. Kohlberg and Candee argue that on both philosophic and psychological grounds use of social relativism is invalid [1]. Moral judgment has been identified by Kohlberg at the highest stage, as having “universalizable intent and that agreement and consensus are necessary and desirable features of moral discourse” (p.46) [37]. “Even if following the moral method does not lead to substantive agreement, critical elements are impartiality,… universalisability, prescriptivity, reversibility and generality”(p.524) [1]. While Kohlberg focussed on the justice principle, he acknowledges that “in many situations, consideration of principle, even those posed as conflicting principles by moral philosophers, like the utilitarian principle of welfare and the Kantian principle of justice, are in agreement about particular situations. The empirical support for this claim is that principled Stage 5 thinkers [those who use UP reasoning] indeed do agree upon which action is right in many conflicting situations”(p. 509) [1]. Rest also argued for a broadening of the highest level of moral development to incorporate all moral ideals which are constructive, sharable and not self-serving at the expense of others [4]. As this study suggests that the majority of tertiary students from both animal and non-animal disciplines, in Australia at least, do prioritise and apply universal principles to animal ethics issues, even more than to human ethics issues, the challenge for educators is to enable these high levels of moral judgment to be acknowledged and applied to address animal ethics issues, and embed them in professional and legal practice.

It is possible that since all course groups in this study had higher levels of UP reasoning on animal compared with human ethics issues, the higher levels may be due to the subject matter or the test instrument. In contrast to the human ethics issues in the DIT, all three animal ethics scenarios involved vulnerable animals in potentially severely harmful situations. It is also possible that compassion, an empathic moral sentiment,[38] may have prompted more principled reasoning than in the human scenarios. Compassion has been identified as having cognitive process involving evaluation of the subjects’ situation as serious, undeserved and an important part of one’s own scheme of ends and goals [39].

Differences in PI and MN reasoning on animal and human ethics issues between students in different programs may reflect demographic differences. Arts students’ higher PI reasoning than animal science and medical students on animal ethics issues, and most other groups except Vet Tech on human ethics issues, and lower MN reasoning than most other groups on animal ethics issues except animal science may be due to having the smallest proportion of students with a previous degree and the youngest age group. Many studies have shown that education and to a lesser extent age are positively correlated with moral judgement [4]. As Arts students had completed most of an ethics course, it is somewhat surprising that they had more PI reasoning. Students of liberal arts programs have been found to have higher moral reasoning growth than those in vocationally oriented higher education courses perhaps due to the focus on “bringing students into contact with a highly diverse range of facts and views about the world… which address the complexities and dilemmas that arise as different people seek to live cooperatively in the world (p.28)” [40]. However, overall there was relatively little PI, compared with MN and UP, reasoning and these students were in the first year of their Arts program.

Medical students higher use of MN reasoning on animal ethics issues than Arts, Vet Sci, Vet Tech and Anim Sci students may be the result of other demographic factors. Higher MN scores were identified in males than females on animal ethics issues in this study, and there was a trend for previous degree to also have a positive effect on MN scores. The Med student group had the highest proportion of male students, particularly compared with Vet Tech, Anim Sci, and Vet Sci groups, and to a lesser extent, the Arts group. As well, all Med students had a previous degree, compared with very low proportions with previous degrees in the Arts, Anim Sci and Vet Tech student groups and a low proportion in Vet Sci. Although education level is the most important factor in developing moral judgment [4], the effects of different programs and colleges have also been identified [40]. Medical and veterinary science students had similar levels of UP reasoning to other groups on animal ethics issues, but higher UP reasoning on human ethics issues. It is possible therefore that the previous mainly science programs were not developing principled moral reasoning in relation to animal ethics, as much as human ethics issues.

Higher MN and lower UP reasoning of students whose primary language was not English on animal ethics issues, but not on human ethics issues, aligns with an earlier study of Australian first year veterinary students indicating that students whose primary language was not English were less strongly concerned about how animals are treated in the Australian community and were more uncertain that they had experienced moral distress [18]. Students who place more importance on maintaining existing social and legal norms are likely to be less conflicted and therefore less concerned about, or perhaps even unaware of, inconsistencies in current social and legal practices related to the treatment of animals. Cultural differences in attitudes toward animal use [41, 42] have previously been identified. As this study involved students from one Australian university, further research is needed to determine if students’ moral judgment development is similar in other universities and in other cultural settings. International research into the relationship of field of university study to attitudes toward animals identified that agriculture students (agriculture, forestry, fishery and veterinary) were more accepting of killing animals, unnatural practices on animals (such as genetic selection and modification which change their natural state) and animal experimentation; humanities and arts students (religion, theology, languages, history, archaeology, philosophy, fine and performing arts) were less accepting of unnatural practices on animals and animal experimentation than students of other disciplines [43].

This study further validates the VetDIT-V2 as a measurement tool due to the positive correlations between scores for the animal scenarios, which were strongest for MN and UP scores, and the correlations between the combined scores for the three animal and three human scenarios (though low). The greater variation within animal scenarios between PI, MN and UP scores, with PI scores having greater variation, than MN which were greater than UP scores, compared with similar variations between PI, MN and UP scores within human scenarios, was most likely due to the very low numbers of students who selected PI and the much greater number of students who selected UP items as important in the animal scenarios.

## Conclusions

This comparison of first year Vet Sci, Vet Tech, Anim Sci, Med and Arts students’ moral judgment on animal and human ethics issues using the VetDIT-V2 suggests greater use of universal principles on animal ethics issues than human ethics issues, regardless of whether the students have chosen animal-related professions. Students used minimal PI reasoning on animal ethics issues, less than on human ethics issues. Use of MN reasoning was similar on both animal and human issues, and reflected the levels used by a mixed sample of US students at equivalent educational levels. Medical students, all of whom had a previous degree and the largest proportion of male students, used more maintaining norms reasoning than any other group. On animal ethics issues, male students and students whose English was not their primary language used more MN and less UP reasoning. On human ethics issues, males used more PI and less UP reasoning and UP scores increased with students’ age. This study further validates the VetDIT-V2 as a tool for assessing and comparing students’ moral judgment development. The high importance given to principled reasoning by all first year student groups in this study suggests that for many students one of the key components enabling moral action is already well-developed. This has implications for animal-related professions and education programs to build on students’ moral judgment and develop capacity to address animal ethics issues, and thus also help avoid moral distress and a disillusioned professional experience.

## Supporting Information

S1 Appendix VetDIT Version 2. (PDF)
