# Supplementary material for: Differences in Moral Judgment on Animal and Human Ethics Issues between University Students in Animal-Related, Human Medical and Arts Programs
Source: PLoS One. 2016 Mar 2;11(3):e0149308. doi: 10.1371/journal.pone.0149308 (PMC4774973; doi:10.1371/journal.pone.0149308)
Supplement: S1 Appendix — (PDF) [file pone.0149308.s001.pdf]

THE UNIVERSITY OF QUEENSLAND AUSTRALIA

CAWE

VetDIT V2

Copyright JM Verrinder, R Ostini & CJC Phillips

Centre for Animal Welfare and Ethics, University of Queensland, Australia

Based on DIT-2 by J Rest & D Narvaez

If you consent to this questionnaire being used for research purposes, please record a self-established research code using the formula below:

Research Code *: ____________

** Formula for Code: The **year** of your birth, the **month** of your birth, and the **first 4 letters** (or 2-3 letters if the name is shorter) of your **first pet's name** e.g. 199206SMOK. If you have never had a pet, use the first 2-4 letters of your first best friend's name.*

Instructions

This questionnaire is concerned with how you define the issues in a social problem. Three animal scenarios are presented. After each scenario are twelve questions representing different issues that might be raised by the problem. In other words, the questions/issues raise different ways of thinking about what is important in making a decision about the social problems. You will be asked to **rate and rank** the questions in terms of **how important** each one seems to you.

Here's an example: Imagine that you are able to vote for a candidate for Prime Minister/President of your country. Before you vote, you are asked to rate how important some issues are in making up your mind about which candidate to vote for, using a rating scale of 1 to 5 (below) and circling the chosen level of importance for each issue. Assume that you thought that Question 1 was of great importance, Question 2 had some importance, Question 3 had no importance, Question 4 had much importance, and Question 5 had much importance. Using the following scale, you would record this as:

(1) = Great importance (2) = Much importance (3) = Some importance (4) = Little importance (5) = No importance

(1) 2 3 4 5 — 1. Financially has the current government made me better off?

1 2 (3) 4 5 — 2. Does the candidate have a superior moral character?

1 2 3 4 (5) — 3. Which candidate is the best looking?

1 (2) 3 4 5 — 4. Which candidate would make the best world leader?

1 (2) 3 4 5 — 5. Which candidate has the best ideas for our country's internal problems, like crime and health care?

Further, the questionnaire will ask you to rank the questions in order of importance. In the space below, the numbers represent the issue number. For example based on your ratings above, you may choose the most important issue to consider as Question 1. *Financially am I better off*; the second most important issue as Question 5. *Who has the best ideas for internal problems* ...; the third most important issue as Question 4. *Who would make the best world leader*; and the fourth most important issue as Question 2. *Who has a superior moral character*, as follows:

Most important issue	(1) 2 3 4 5 6 7 8 9 10 11 12 (*12 issues are presented for each social problem*)
Second most important issue	1 2 3 4 (5) 6 7 8 9 10 11 12
Third most important	1 2 3 (4) 5 6 7 8 9 10 11 12
Fourth most important issue	1 (2) 3 4 5 6 7 8 9 10 11 12

***Note**: Some of the items may seem irrelevant to you (as in Item 3) or not make sense to you, in that case, rate as "No" importance, and do not **rank** the item.*

In addition you will be asked to state your preference for what action to take in a story i.e. (1= strongly favour some action, 2= can't decide, 3 = strongly oppose the action. For example:

1. Should vote for Candidate A 2. Can't Decide (3) Should not vote for Candidate A

PLEASE BEGIN

Scenario 1 - Request to Euthanize a Healthy Dog

A woman brings her lively five year old kelpie/cattle cross dog in to see a veterinarian, Dr Benjamin, for euthanasia. She says she is moving into an apartment with her boyfriend who doesn't like the dog, and pets aren't allowed in the apartment building. Besides this, the dog is too active for her and is barking all the time. The veterinarian asks if she has tried to put the dog up for adoption, but she replies that the local pound already has too many working dogs and they would probably euthanize it anyway. She simply wants the dog humanely destroyed and, if the veterinarian doesn't euthanize it, her boyfriend will shoot it. Dr Benjamin wonders what to do.

A. What action do you favour the veterinarian taking? (mark one below):

1. Should euthanize the dog	2. Can't decide	3. Should not euthanize the dog

B. Rate the following 12 issues in terms of importance (1-5): (1 = Great importance, 2 = Much importance, 3 = Some importance, 4 = Little importance, 5 = No importance)

Great (1)* to *No (5) importance

1 2 3 4 5 1. Should the veterinarian support the owner's legal right to euthanize the dog?

1 2 3 4 5 2. Should the veterinarian risk losing a client by refusing to euthanize the dog?

1 2 3 4 5 3. Does the dog have a right to life?

1 2 3 4 5 4. Would the Australian Veterinary Journal be interested in an article on this?

1 2 3 4 5 5. Would refusal to euthanize cause a confrontation with the owner and the boyfriend?

1 2 3 4 5 6. What action would be supported by the veterinary profession?

1 2 3 4 5 7. Should the veterinarian secretly rehome the dog out of respect for its life?

1 2 3 4 5 8. Is it more caring to argue for the dog's life or accept the owner's decision?

1 2 3 4 5 9. Should the veterinarian tell the client to find another veterinarian to euthanize the dog?

1 2 3 4 5 10. What do most veterinarians in Australia do in this situation?

1 2 3 4 5 11. Should the veterinarian weigh up the possible consequences to all concerned of euthanizing the dog?

1 2 3 4 5 12. Does the veterinarian have the time to consider this issue in his/her busy day?

C. Rank which issue is most important (by circling the question number below):

Most important issue to consider *1 2 3 4 5 6 7 8 9 10 11 12*
Second most important issue to consider *1 2 3 4 5 6 7 8 9 10 11 12*
Third most important issue to consider *1 2 3 4 5 6 7 8 9 10 11 12*
Fourth most important issue to consider *1 2 3 4 5 6 7 8 9 10 11 12*

Scenario 2 - Pig Husbandry

Dr Jones, a veterinarian, examines a sick pig at a large scale piggery that she visits once or twice per year. It is emaciated, has diarrhoea, and is pregnant. There are approximately 20 other pigs that the veterinarian can see are also in a state of serious ill-health. The owner says he is having a tough time in the current economic climate. He wants the veterinarian only to treat the one pig. The quality of animal husbandry on the farm seems to have deteriorated over the years, despite the veterinarian offering suggestions. Dr Jones wonders whether she should report the owner to the appropriate authority.

A. What action do you favour the veterinarian taking? (circle the action)

1. Report the farmer to the authorities	2. Can't decide	3. Do not report the farmer to the authorities

B. Rate the following 12 issues in terms of importance (1-5): (1 = Great importance, 2 = Much importance, 3 = Some importance, 4 = Little importance, 5 = No importance)

Great (1)* to *No (5) importance

1 2 3 4 5 1. Is it a veterinarian's professional role to make this judgement?

1 2 3 4 5 2. Could the veterinarian best help by treating all the pigs for a lower price?

1 2 3 4 5 3. Will other farmers want to employ the veterinarian if she reports this farmer?

1 2 3 4 5 4. Will there be more benefit than harm, if the farmer is reported?

1 2 3 4 5 5. Is it within the bounds of accepted practice to lose some animals in any large scale animal production system?

1 2 3 4 5 6. Is pork more popular than chicken meat?

1 2 3 4 5 7. Should the veterinarian's decision be based on what the AVA Code suggests?

1 2 3 4 5 8. Is it worth the trouble of reporting this one farmer?

1 2 3 4 5 9. Is it unlikely for a prosecution to be successful in a case like this?

1 2 3 4 5 10. Will the farmer blame the vet?

1 2 3 4 5 11. Will the veterinarian be upset if she reports the farmer?

1 2 3 4 5 12. Do the pigs have a right to treatment?

C. Rank which issue is most important (by circling the question number below):

Most important issue to consider *1 2 3 4 5 6 7 8 9 10 11 12*
Second most important issue to consider *1 2 3 4 5 6 7 8 9 10 11 12*
Third most important issue to consider *1 2 3 4 5 6 7 8 9 10 11 12*
Fourth most important issue to consider *1 2 3 4 5 6 7 8 9 10 11 12*

Scenario 3 - Breeding modification in confinement agriculture

In large-scale commercial egg production housing systems, laying hens often engage in feather pecking, which leads to damage to plumage, flesh wounds, and, in the worst cases, a risk of cannibalism. A common way of reducing these effects is removing the tips of the beaks of day old chickens. Another possible approach involves breeding congenitally blind hens. Research with blind adult hens at commercial stocking densities indicated these hens were physically and socially less active, with less feather pecking, less comb damage, and higher egg output, than sighted birds, whilst maintaining similar body weight. In another study, blind chickens up to six weeks old sat and preened more, did less environmental pecking, showed reduced behavioural synchrony and group aggregation, and lower body weight, and exhibited a number of abnormal behaviours, suggesting they may be more stressed, and likely to miss positive experiences of moving easily, social interaction, and finding food. A veterinarian, Dr Vivardi, is asked to provide professional advice regarding whether a proposed development plan, to breed congenitally blind chickens to assess welfare and productivity on a commercial scale, should proceed.

A. What action do you favour the veterinarian taking? (circle the action)

1. Should advise the research proceeds	2. Can't decide	3. Should advise against the research

B. Rate the following 12 issues in terms of importance (1-5): (1 = Great importance, 2 = Much importance, 3 = Some importance, 4 = Little importance, 5 = No importance)

Great (1) **to** ***No (5) importance***

1 2 3 4 5 1. Are the public likely to abuse the veterinarian if he/she advises the research proceeds?

1 2 3 4 5 2. Is this research in line with veterinarians' accepted standards?

1 2 3 4 5 3. Is it important to consider whether the benefits of less stress to the birds outweigh the harm of taking away one of their natural features?

1 2 3 4 5 4. Which decision is better for the future of the egg farming industry?

1 2 3 4 5 5. Is it fair to manipulate animals to fit production systems?

1 2 3 4 5 6. Would the vet be criticised professionally for having an emotional unscientific reaction if he/she opposed this?

1 2 3 4 5 7. Is it disrespectful to interfere with the “wholeness” of a bird?

1 2 3 4 5 8. Is this any different from breeding practices which have been used for many years to modify characteristics of farm animals?

1 2 3 4 5 9. Are the natural sciences better than the pure sciences?

1 2 3 4 5 10. Is recommending the breeding of blind chickens what a good person would do?

1 2 3 4 5 11. Will this project provide interesting work for the veterinarian if it goes ahead?

1 2 3 4 5 12. If it is legal, is there any reason not to genetically modify farm animals?

C. Rank which issue is most important (by circling the question number below):

Most important issue to consider *1 2 3 4 5 6 7 8 9 10 11 12*
Second most important issue to consider *1 2 3 4 5 6 7 8 9 10 11 12*
Third most important issue to consider *1 2 3 4 5 6 7 8 9 10 11 12*
Fourth most important issue to consider *1 2 3 4 5 6 7 8 9 10 11 12*

<u>Demographic Information</u>:

Please circle the most appropriate response or record your answer as required:

1. Current Age: _______________

2. Sex: Male   Female

3a. Degree currently enrolled in: ____

b. Previous university degree(s): Yes   No

c. If yes, please identify degrees completed(s):

____

4. Is English your primary language? Yes   No

5. Rate the extent to which you have had experience with the following groups of animals:

1 = Very great extent; 2 = Great extent; 3 = Some extent; 4 = Minimal extent; 5 = Never

a. Companion animals   1 2 3 4 5

b. Farm animals   1 2 3 4 5

c. Horses   1 2 3 4 5

Thank you for completing this questionnaire.

Do you have any comments on any aspects of the questionnaire, including any difficulties you had, suggestions for improvement, or just general comments? We really appreciate your feedback.

____

____

____

____

____
